# Vaccines in cancer treatment and prevention: the time is now

**DOI:** 10.1172/JCI195673

**Published:** 2025-07-01

**Authors:** William Becker, W. Kimryn Rathmell

**Affiliations:** 1 National Institute of Diabetes and Digestive and Kidney Diseases, National Institutes of Health, Bethesda, Maryland, USA.; 2 Ohio State University, Columbus, Ohio, USA.

## Vaccines as cancer prevention agents

The immune system is finely tuned to protect us from foreign pathogens but can also detect new antigens expressed during the development of cancer. Cancer is a formidable opponent, one in which host cells transform and flexibly adapt to evade and dampen immune responses. By 2050, the global predicted cancer burden is a staggering 35 million new cases a year. Drawing from the lessons learned in harnessing the immune system to combat infectious disease, vaccines are a tool that present great potential to reduce the global cancer burden dramatically. An emerging horizon is therapeutic vaccines for cancer, directing the immune system in a more precise manner than current therapies. Here, we discuss the current state of vaccines for cancer prevention, the ongoing efforts to develop vaccines for cancer therapy, and remaining challenges for the field.

Vaccines play an important role in preventing cancer. Hepatocellular carcinoma (HCC) accounts for 90% of all primary liver cancer cases, ranking third in mortality among all cancers, and over half of all HCC cases are attributable to cirrhosis associated with chronic infection with hepatitis B virus (HBV) (1). The HBV vaccine, available since the 1980s, will prevent an estimated 38 million deaths among people born between 2000 and 2030 and save more than $120 billion over the same time period, making vaccination the best and cheapest way to prevent HBV-related HCC (2). Similarly, widespread adoption of vaccines targeting human papillomavirus (HPV) has been shown to effectively reduce the incidence of several cancers and has the potential to virtually eliminate risk for cervical cancer (3). Roughly one sixth of all human cancers result from only seven viruses, and tackling these viruses through vaccination represents an enormous opportunity to reduce cancer incidence worldwide — cheaply, and for life (4). This scale of effect cannot be understated.

Vaccines also offer a preventative opportunity for cancers that are not virally mediated. As an example, the genetic condition known as Lynch syndrome predisposes individuals to an array of cancers, all resulting from predictable genetic defects in DNA mismatch repair genes. Early phase studies are underway for individuals with Lynch syndrome to test if vaccines that target defined common neoantigens (new antigens that form in tumors due to genetic mutations) can prevent or delay cancer (5).

Notably, directing immunity with vaccines does not need to be limited to cancer prevention. With the available technology, vaccines can steer the immune system against cancer cells in ways that enable both improved disease control and reduced toxicity by introducing a level of specificity that is urgently needed. The promise of this approach reflects the culmination of a long history of augmenting our immune system to treat cancer.

## The long saga of immunity and cancer

The idea of mobilizing the immune system to combat cancer goes back to ancient times. In 2700 BCE, the Egyptian physician Imhotep reportedly induced infections on tumors as a treatment strategy. Furthering this concept, interventions such as the Bacillus Calmette-Guérin (BCG) vaccine acts as a nonspecific agent eliciting an immune reaction at the bladder mucosa applied in nonmuscle invasive bladder cancer were developed and remain a standard of care today (6). These and other early tools were limited, however, by the lack of antigen specificity. The discovery of tumor-specific antigens hailed a coming-of-age for precision therapy by enabling cancer vaccines against proteins found specifically on cancer cells. Despite astounding safety profiles for cancer vaccines, first-generation designs and the underestimation of cancer’s fierce resistance to an immune response resulted in a series of disappointing attempts to show substantial clinical response (7). Renewed hope for cancer vaccines rebounded with the approval of the first dendritic cell-based vaccine immunotherapy in 2011, known as sipuleucel-T. But enthusiasm was premature; sipuleucel-T can prolong survival in subsets of prostate cancer patients (8), but the expense and modest extension in survival in individual patients limited the impact.

Ultimately, what may create the largest opportunity for vaccine therapy to emerge as a game changer is the rise in immune checkpoint inhibitors (ICIs) (9). ICIs function by tipping the delicate internal balance of CD8^+^ T cells from self-tolerance towards cancer elimination. ICIs are widely acclaimed for long-lasting responses that can occur in many cancer patients, which drives a population-level impact on outcome; however, individual responses are unpredictable and variable. Researchers are now pursuing strategies that improve efficacy or expand eligibility for these treatments by reducing adverse side effects due to immune-related toxicities (10). In the age of personalized medicine, immunotherapies should exploit the knowledge gained from infectious disease and autoimmunity research to refine treatment modalities to be safer, more effective, and specific against cancer. Vaccination is an ideal approach to meet this need.

## The synergy between cancer vaccines and immunotherapy

ICIs work best when CD8^+^ T cells are already present in or around a tumor. Not all CD8^+^ T cells are equal, and the best responses occur when the CD8^+^ T cell compartment is ‘stem-like’, armed with the capacity for replication and tumor killing (11). However, tumor cells are fiercely resilient and adapt mechanisms to impair immune invaders. The ongoing DNA damage, epigenetic reprogramming, and unregulated growth create opportunities for tumor evolution while also generating a catalog of neoantigens. These neoantigens arising during the process of tumor development represent potential targets for therapeutic vaccination.

Unfortunately, only a fraction of known neoantigens elicit noticeable T cell responses in tumors, and T cells found in tumors that are specific for neoantigens are often ‘exhausted’, unable to kill the tumor or respond effectively to ICI (12). Fewer than half of the high frequency mutations in cancer are likely to bind the most common HLA-A or B alleles, reducing the likelihood of success for ‘off-the-shelf’ neoantigen vaccines (13). However, genomic features that arise over the course of tumorigenesis, such as expression of endogenous retroviruses, provide opportunities for neoantigen expression (14). Personalized cancer vaccines have been unattainable until now, given the breadth of conserved mechanisms inherent in negative thymic selection to purge autoreactive T cells. Cancer vaccines directed against self-antigens that are modified just enough to be considered neoantigens requires a precision approach to avoid triggering autoimmunity (15), but today’s advances in vaccination technology are poised to make personalized cancer vaccines a reality.

## Improvements in vaccination

The development of RNA-based vaccines represents a major advance for cancer vaccine research and implementation. The groundwork for RNA vaccines lay in decades of basic research, but the global pandemic of COVID-19 spurred accelerated development of vaccine technology. mRNA-based vaccines represent a cheaper, faster, and scalable method for rolling out vaccines over typical peptide-based vaccines — critical for personalized cancer vaccine development. The vaccination of millions of people with mRNA vaccines demonstrated their outstanding safety profile (16). Currently, a number of different strategies and vectors are being pursued to customize vaccine formulation to target specific organs and cancer types (17). These vaccination strategies have the benefit of years of research not only into the improved structures for vaccines but the other factors dictating how we direct T cells.

**Figure 1:**
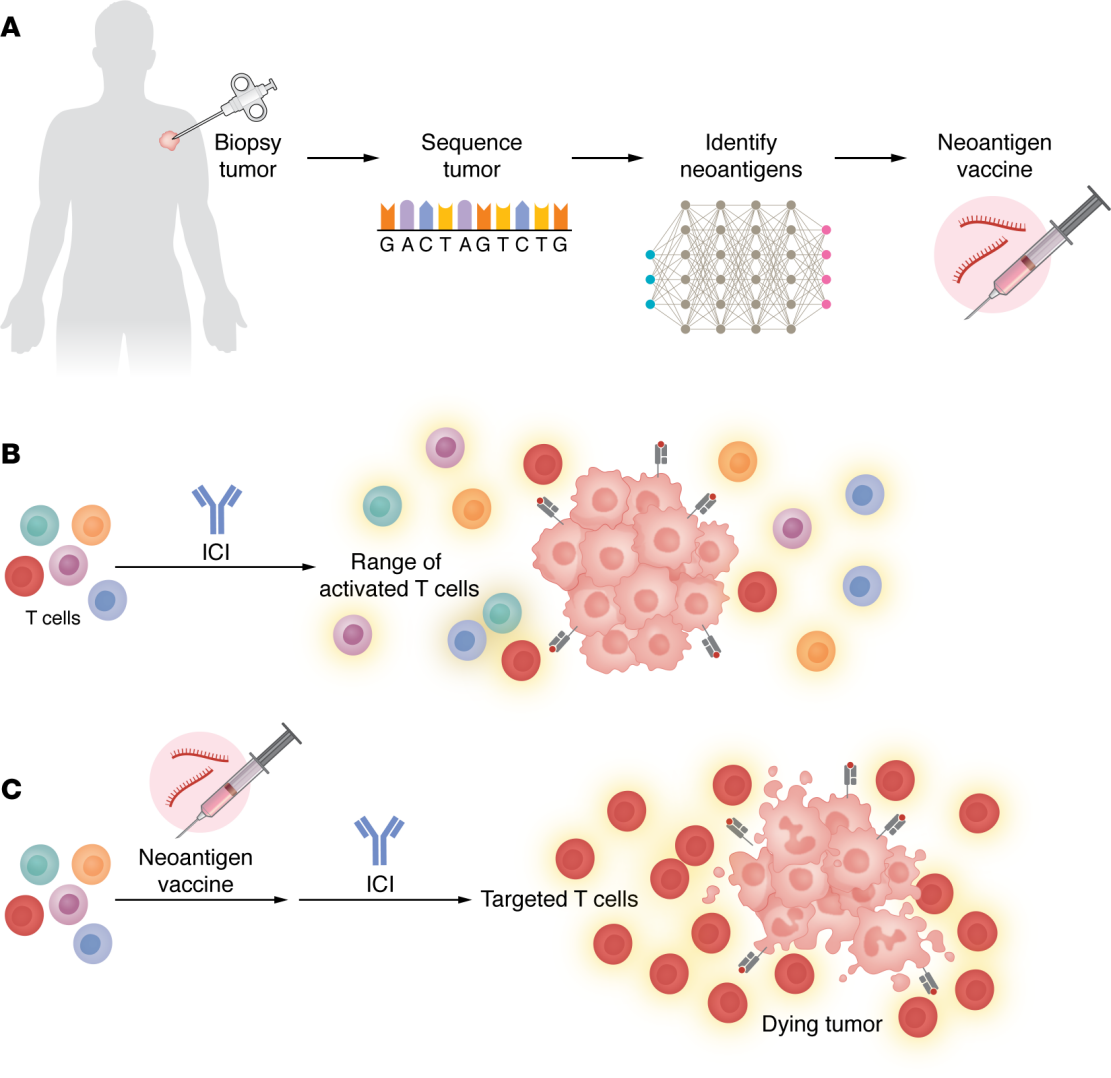
The process and effects of neoantigen vaccines to bring specificity to tumor immune response. (**A**) A flow chart depicting the stages from tumor biopsy to tumor cell sequencing, to neoantigen identification, to mRNA-based neoantigen vaccine administration. (**B**) Antibody immunotherapy activates a range of T cells in the body, with a low proportion of T cells specific to the cancer. (**C**) A neoantigen vaccine can stimulate the patient’s T cells to be optimally targeted against antigens specific to the cancer, and the addition of antibody immunotherapies to the neoantigen vaccine stimulates the response of T cells to attack the cancer.

Vaccination routes, temporal considerations, as well as adjuvants and immunostimulants, all influence vaccine-mediated immune response, efficacy, antigen dose required, and toxicity of the vaccine (18). Altering the current paradigm of intramuscular administration in favor of intravenous (19), intradermal, intranodal, and even mucosal (20) routes has improved durable immunity. Administering an ICI before a cancer vaccine can nullify the curative effect of the combination, so determining the timing of cancer vaccines with ICIs is critical to elicit the best response (21). Ideally, in a future personalized treatment paradigm, vaccination would be integrated into patient care: a patient’s tumor might be surgically removed, tumor DNA sequenced, immunogenic neoantigens determined with in silico and AI models, and vaccines developed efficiently using mRNA technology to administer as a customized immune modulator based on the patient’s own cancer’s genetics (Figure 1). Contrast this with the burdens of radiotherapy, systemic therapies, and adoptive cell therapies where serious adverse events are common, and it’s clear that cancer vaccines represent a potentially safer path towards personalized durable disease control and patient wellbeing.

Although studies testing these strategies are well underway, it is important to be cognizant of the history of cancer vaccines, long promising treatments that failed to meaningfully materialize in the clinic. This perspective was reinforced by two recent large-scale clinical trials for melanoma (22) and glioblastoma (23), where patients receiving vaccines against tumor-associated antigens did not fare better than their control counterparts. Importantly, these studies lacked the critical components of personalized specificity and synergy with ICIs. The future for cancer vaccines must acknowledge lessons of the past to strategically implement advancements in basic science that have primed the long arc of cancer vaccines towards success.

## The emerging promise of personalized cancer vaccines

Several recent studies have tested the combination of cancer vaccines with ICI for secondary prevention in patients with resected tumors. In resected high-risk cutaneous melanoma, patients received an mRNA-based neoantigen vaccine with or without ICI. At 2 years, the vaccine-plus-ICI group had prolonged recurrence-free survival (RFS) and distant metastasis–free survival (DMFS) (24). In high risk ccRCC, patients received a neoantigen peptide vaccine and ICI postoperatively, and at 40 months of follow up, none of the 9 participants had recurred (25). All patients demonstrated an immune response to the vaccine. In pancreatic ductal adenocarcinoma, researchers found that a personalized mRNA neoantigen vaccine, delivered intravenously, led to a longer median RFS, with responders being those generating a T cell response to the vaccine (26), giving investigators both conditions for success and metrics for improving future trials. These studies showed impressive safety profiles and demonstrated a range of tumors for which this technology may offer a substantive advantage. Later metastasis is still the primary cause of lethality for most cancers, and the generation of long-lived T cell clones specific to cancers, as demonstrated in these early studies (26), represents the most reliable way to naturally protect from disease recurrence. Randomized phase II trials for wide range of cancer histologies and neoantigens are underway.

## Conclusions

Decades of progress in infectious disease converging with cancer biology and advanced genomics have primed cancer vaccines to meet the needs of cancer care today. An unparalleled level of precision poises the field to develop therapies that exploit each tumor’s unique molecular mosaic, and the time is now to embrace full bore the promise of this approach. Although established preventative vaccines have experienced waning popularity due to vaccine hesitancy, these agents are highly effective, and their uptake at the population level benefits from the same evidence-based and culturally appropriate interventions that have been applied in the infectious disease prevention setting to widen vaccine acceptance (27). The issue will be not *if* we should be using cancer vaccines, but *how* to implement them for optimal patient outcomes.

We recognize that cancer vaccines won’t cure all cancers. Like all medical innovations, they will evolve through testing and refinement to improve patient outcomes. It is imperative that we continue to support innovation in this area to extend this opportunity to the broadest set of conditions, and that we engage the patient community in this transformation of care to allow for optimal penetration of these tools for cancer. It’s clear that enabling memory T cells to identify and selectively kill cancer cells represents a crucial method — and likely our best hope — for eradicating emerging or existing cancers, heralding the ultimate in precision oncology.

## Version 1 07/01/2025

Electronic publication
